# Functional Polyglycidol-Based Block Copolymers for DNA Complexation

**DOI:** 10.3390/ijms22179606

**Published:** 2021-09-04

**Authors:** Radostina Kalinova, Miroslava Valchanova, Ivaylo Dimitrov, Sevdalina Turmanova, Iva Ugrinova, Maria Petrova, Zlatina Vlahova, Stanislav Rangelov

**Affiliations:** 1 Institute of Polymers, Bulgarian Academy of Sciences, 1113 Sofia, Bulgaria; kalinova@polymer.bas.bg; 2 Department of Material Science and Technology, University “Prof. Assen Zlatarov”, 8010 Burgas, Bulgaria; m.a.valchanova@abv.bg (M.V.); sturmanova@abv.bg (S.T.); 3 Institute of Molecular Biology, Bulgarian Academy of Sciences, 1113 Sofia, Bulgaria; mhristova84@abv.bg (M.P.); vlahova94@gmail.com (Z.V.)

**Keywords:** gene delivery, non-viral vectors, polyplex formation, polyglycidol copolymers, cationic copolymers, DNA complexation, cell internalization, cytotoxicity

## Abstract

Gene therapy is an attractive therapeutic method for the treatment of genetic disorders for which the efficient delivery of nucleic acids into a target cell is critical. The present study is aimed at evaluating the potential of copolymers based on linear polyglycidol to act as carriers of nucleic acids. Functional copolymers with linear polyglycidol as a non-ionic hydrophilic block and a second block bearing amine hydrochloride pendant groups were prepared using previously synthesized poly(allyl glycidyl ether)-b-polyglycidol block copolymers as precursors. The amine functionalities were introduced via highly efficient radical addition of 2-aminoethanethiol hydrochloride to the alkene side groups. The modified copolymers formed loose aggregates with strongly positive surface charge in aqueous media, stabilized by the presence of dodecyl residues at the end of the copolymer structures and the hydrogen-bonding interactions in polyglycidol segments. The copolymer aggregates were able to condense DNA into stable and compact nanosized polyplex particles through electrostatic interactions. The copolymers and the corresponding polyplexes showed low to moderate cytotoxicity on a panel of human cancer cell lines. The cell internalization evaluation demonstrated the capability of the polyplexes to successfully deliver DNA into the cancer cells.

## 1. Introduction

The basic principle of gene therapy consists of exogenous delivery of foreign genetic material into target cells to promote a therapeutic effect in patients by adding, replacing, or editing a gene that is absent or abnormal or by knocking out the expression of a mutated gene [1,2,3]. It has the potential to shift the way numerous acquired and hereditary genetic diseases are managed and treated, in which the delivery of nucleic acids is crucial. Engineered viruses have been the first gene delivery agents. Although efficient, they appeared quite risky for the patient because of their inherent immunogenic nature, severe side effects, and high toxicity, which hampered their clinical applications [4,5,6]. The documented danger of the viral carriers has motivated the exploration of gene delivery alternatives based on lipids, polymers, and inorganic materials [7,8], which are safer, less pathogenic, and less immunogenic. Among them, polymer-based vectors have attracted intensive research interest [9,10,11,12,13]. In addition to the potential safety benefits, polymers offer great structural and chemical versatility for manipulating the physicochemical properties, access to large scale production, batch to batch reproducibility, large nucleic acid loading capacity, stability upon storage, low cost of treatment, all of them being advantageous for such a complex and complicated process such as gene therapy.

The polymeric vectors typically exploit the anionic nature of DNA to drive complexation via electrostatic interactions. Cationic polymers such as polyethyleneimine (PEI), poly(L-lysine) (PLL), poly(2-(dimethylamino)ethyl methacrylate) (PDMAEMA), and polyamidoamine, as well as natural polymers such as chitosan, dextran, and pullulan, are able to condense the bulky structure of DNA into nanosized complexes (polyplexes) [14,15]. The condensation also ensures neutralization of the negatively charged phosphate backbone of DNA and its protection from both extracellular and intracellular nuclease degradation. Ideally, DNA should remain active and capable of transfecting cells. However, the significant cytotoxicity of the cationic polymers, as well as the fast recognition by the reticuloendothelial system (RES) and rapid blood clearance of the polyplexes, are still an issue. The uptake by the RES can be suppressed and cytotoxicity reduced by incorporation of hydrophilic, flexible polymer moieties/segments. Besides, they can enhance the colloidal stability, favorably influence the physicochemical properties, and introduce functionalities that can be helpful in overcoming biological barriers to gene delivery. However, all those benefits of incorporation of additional moieties are frequently negated by the ubiquitous reduction in efficiency so that the delicate balance between apparently conflicting functions and properties should always be sought.

Building from the success of PEGylation of, firstly, proteins and later drugs and nanoparticles, it is not surprising that poly(ethylene glycol) (PEG) has been the most commonly employed for modification of cationic polymer-based polyplexes [16,17,18]. The role of PEG coatings is commonly associated with increasing solubility, imparting steric stabilization and stealth properties, preventing aggregation, decreasing cytotoxicity and immunogenicity, reducing opsonization and phagocytosis, and prolonging systemic circulation time. PEG coatings were also utilized for overcoming various biological barriers to efficient drug and gene delivery as described elsewhere [19,20,21,22].

Closely related to PEG and imparting essentially the same properties are polymers of oligo(ethylene glycol) methacrylates (OEGMAs). OEGMAs are easily polymerizable by controlled radical polymerizations allowing for a broad range of linear and non-linear random and block copolymers, including copolymers with polycationic moieties, exhibiting potential in gene delivery [23,24,25,26]. In contrast to PEG, the macromolecules of POEGMAs are considerably thicker and bulkier. They are composed of hydrophilic side chains of oligo(ethylene glycol) and a hydrophobic methacrylate main chain, which imparts amphiphilic properties: POEGMAs with side chains length shorter than nine oxyethylene units exhibit thermoresponsive behavior in aqueous solution [27,28,29].

A convenient source for obtaining linear PEI by acidic or basic hydrolysis is the poly(2-oxazoline)s (POx) [30,31,32,33,34,35]. The latter polymers can be produced by living cationic ring-opening polymerization of 2-oxazolines, which provides access to a variety of well-defined polymers [30,31]. POx are versatile with a range of end-group and side-chain functionalization. Their properties, in particular water solubility, can be easily tuned by varying the side chain of the 2-oxazoline monomer: POx with short side chains (methyl, ethyl, propyl, iso-propyl) are water-soluble and/or thermoresponsive polymers, whereas longer aliphatic or aromatic substituents result in hydrophobic polymers. POx exhibit excellent biocompatibility with various biological systems and represent an attractive platform for the development of biomaterials [31,32,36]. Those with short alkyl side chain substituents are considered promising alternatives to PEG. Of particular importance is the partial hydrolysis of POx by which PEI segments in desired quantities and distribution can be introduced [37]. The resulting POx-PEI copolymers combine PEI moieties capable of binding nucleic acids with hydrophilic and/or thermoresponsive, biocompatible, and biologically tolerant POx moieties [37].

**Scheme 1:**
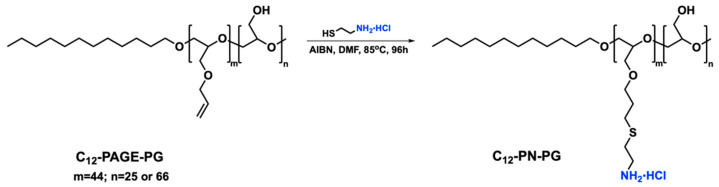
Synthetic route to functional block copolymers with pendant primary amine hydrochloride groups.

Exploiting the concept of seeking a proper balance between safety and efficiency, we suggest another hydrophilic, non-ionic, and biologically tolerant polymer, which shows promise for the development of biomaterials and for other biomedical applications: linear polyglycidol (PG). PG is structurally similar to PEG: it possesses a polyether backbone (see Scheme 1 for the chemical structure of PG), in which, unlike PEG, pendant hydroxymethylene groups in each repeating monomer unit are introduced. The introduction of numerous hydroxyl groups enriches the possibilities for post-polymerization modification, but it is also manifested in the alteration of the properties of PG as compared to those of PEG. The hydroxyl groups were reported to promote the formation of a strongly hydrogen-bonded PG layer around hydrophobic domains of PG-based copolymers and non-phospholipid PG conjugates, which resulted in drastic changes in the aqueous solution properties and biological performance of these materials [38,39,40,41,42,43]. The present study aimed at evaluating the potential of copolymers based on linear polyglycidol to act as carriers of nucleic acids. From the library of PG-based copolymers studied earlier [38,41,42,43], we selected diblock copolymers of polyglycidol and poly(allyl glycidyl ether) (PAGE) bearing C_12_ hydrocarbon residue (hereinafter, C_12_-PAGE-PG). The PAGE moieties were modified by a thiol-ene click reaction to introduce a strong positive charge at each repeating unit. We set out to characterize the polyplex particles that are formed upon electrostatic interactions with DNA and evaluate the biocompatibility and ability to introduce DNA into cells.

## 2. Results and Discussion

### 2.1. Synthesis and Characterization of Amine-Functionalized Block Copolyethers

The copolymer precursors, abbreviated C_12_-PAGE-PG25 and C_12_-PAGE-PG60, were synthesized by sequential anionic ring-opening polymerization of allyl glycidyl ether (AGE) and ethoxyethyl glycidyl ether (protected glycidol) followed by removal of the protective ethoxyethyl groups as described elsewhere [42]. The hydrophobic hydrocarbon residue was introduced by the initiation of the polymerization with partially deprotonated dodecanol. The high degree of control over the polymerization of the two monomers, as well as the quantitative removal of the protective groups, allowed precise adjustment of the copolymer composition. The two copolymer precursors comprised a hydrophobic dodecyl residue attached to a block of PAGE with a fixed degree of polymerization and a block of PG of differing degrees of polymerization corresponding to PG contents of 25 and 60 mol % for C_12_-PAGE-PG25 and C_12_-PAGE-PG60, respectively (see Scheme 1 and Table 1 for the chemical structure, compositions and degrees of polymerization). The allyl functionality present in the PAGE block was exploited for orthogonal modification to introduce pendant primary amine hydrochloride groups. The synthetic procedure towards functionalization of the PAGE block is presented in Scheme 1.

**Figure 1:**
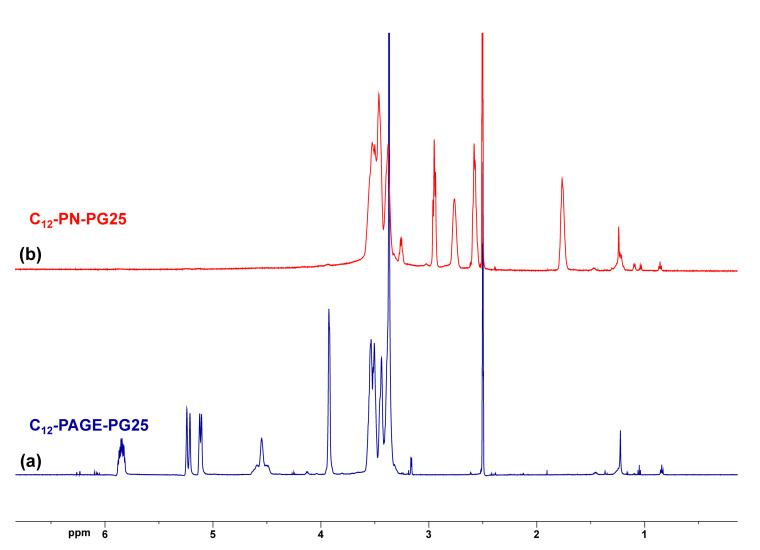
^1^H NMR (600 MHz) spectra in DMSO-d_6_ of (**a**) amphiphilic block copolymer with alkene pendant groups (C_12_-PAGE-PG25) and (**b**) amine-functionalized block copolymer C_12_-PN-PG25.

The radical addition of 2-aminoethanethiol hydrochloride (AET·HCl) to the allylic double bonds was performed in DMF in the presence of AIBN. A 5-molar excess of the amine-functional thiol in hydrochloride form was used, and an increased temperature of 85 °C for an extended period of time was applied to ensure the complete modification. The quantitative conversion of the pendant alkene groups from the PAGE block was confirmed by ^1^H NMR spectroscopy (Figure 1). The signals at 5.84, 5.24, and 5.12 ppm characteristic for the three protons of the allylic double bond, as well as those at 3.93 ppm corresponding to the methylene protons next to the double bond completely disappeared from the ^1^H NMR spectrum in DMSO-d_6_ of the purified product (Figure 1b). Moreover, new resonances at 1.76, 2.58, 2.76, and 2.95 ppm attributed to the methylene protons of the pending amine-terminated side groups appeared. Interestingly, the resonance for the hydroxyl-group protons from the polyglycidol block of the precursor copolymer that is clearly visible at 4.55 ppm in its spectrum (Figure 1a) also completely disappeared in the amine-modified copolymer’s spectrum. These groups are not supposed to undergo any transformations during the modification step. Moreover, there are no additional resonances in the product’s NMR spectrum indicating such transformations. Actually, the formed amine-functionalized copolymers are extremely hygroscopic due to the presence of amine hydrochloride side groups on each repeating unit of the modified PAGE blocks leading to increased water content in the samples prepared for NMR analyses. As a result of the rapid exchange between OH protons from the polyglycidol block and H_2_O in the sample, they are not detectable in the NMR spectrum. Similar observations were reported in an ^1^H NMR study of poly(vinyl alcohol)/DMSO-d_6_/H_2_O system [44]. The block copolymer precursors’ and the amine-functionalized products’ characteristics are listed in Table 1.

### 2.2. Self-Association of the Amine-Functional Block Copolymers and DNA Condensation

**Figure 2:**
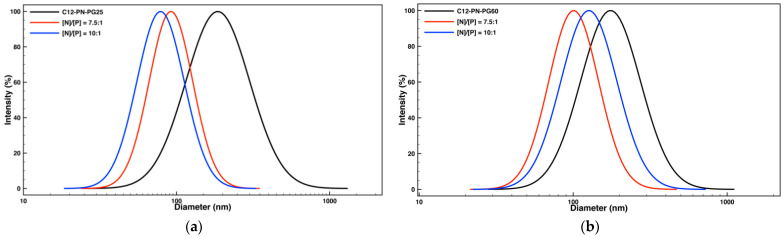
Size-distribution curves from DLS measurements of aqueous dispersions of the block copolymer C_12_-PN-PG25 (**a**) and C_12_-PN-PG60 (**b**) and their polyplexes with DNA at [N]/[P] ratios of 7.5:1 and 10:1.

By introducing amine functionality in the C_12_-PAGE-PG copolymers, reaction sites for electrostatic interactions with nucleic acids are created. These interactions drive the complexation between the modified copolymers (C_12_-PN-PG) and DNA, thereby condensing the bulky structure of the latter to appropriate size for cell internalization, neutralizing its negative charges, and protecting from nuclease degradation. Upon the modification, the initial moderately hydrophobic PAGE block was converted into a strongly hydrophilic one. However, despite the introduction of a large number of densely grafted, highly hydrophilic amine hydrochloride side groups, the amphiphilicity of the C_12_-PN-PG copolymers was not lost: in aqueous solution, the formation of large (175–185 nm) aggregates was detected (Figure 2, Table 1). These aggregates were comparable or larger than the particles that the parent C_12_-PAGE-PG copolymers formed [42] and of considerably broader particle size distributions (Table 1). Furthermore, well-defined, highly contrasted, well-separated, and high electron density objects were not observed by TEM. These findings implied the formation of multichain, loose, and seemingly, not well-defined aggregates. They are held together by two attractive interactions: hydrophobic interactions between the dodecyl residues, albeit weakened by the introduction of numerous hydrophilic protonated amino side groups, and hydrogen bonding via the hydroxyl groups from the PG moieties [39]. The practically identical values of the ζ potential of the aggregates of the two copolymers (Table 1) indicated that the polycation segments are mixed with the PG chains so that the effect of the longer polyglycidol chains of C_12_-PN-PG60 was lost. However, the strongly positive ζ potential of the C_12_-PN-PG aggregates (Table 1), which is considered advantageous as far as complexation with oppositely charged nucleic acids is concerned, is noteworthy.

**Figure 3:**
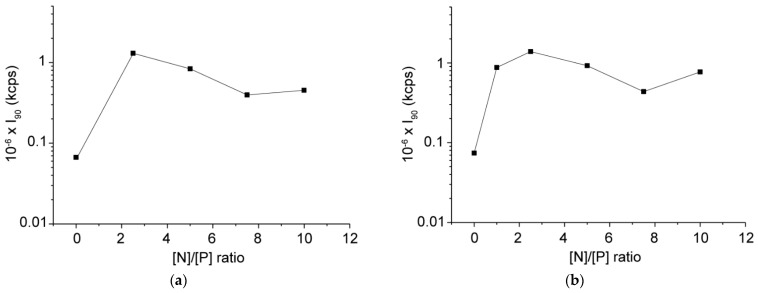
Variations in the scattered light intensity measured at an angle of 90° (I_90_) from dispersions of polyplexes of C_12_-PN-PG25 (**a**) and C_12_-PN-PG60 (**b**) with the [N]/[P] ratio. The lines through the data point are drawn to guide the eye. Data points are at [N]/[P] = 0 refer to the aggregates of the pure copolymers. Standard deviations ±2%.

**Figure 4:**
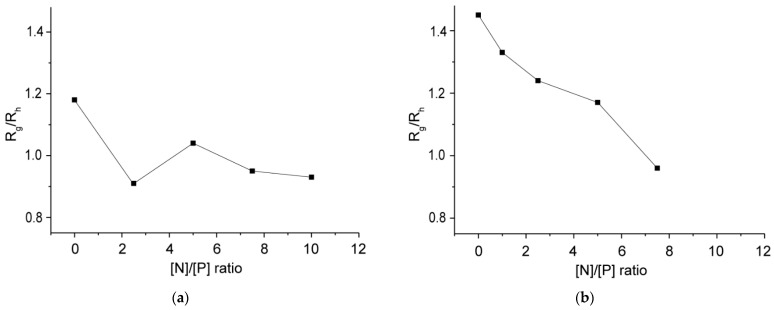
Variations in the quantity R_g_/R_h_ of polyplex particles of C_12_-PN-PG25 (**a**) and C_12_-PN-PG60 (**b**) and DNA with the [N]/[P] ratio. The lines through the data point are drawn to guide the eye. Data points are at [N]/[P] = 0 refer to the aggregates of the pure copolymers. Standard deviations ±2%.

The complexation was performed by slow addition of an aqueous DNA stock solution (100 µg·mL^−1^) to an equal volume of block copolymer dispersions with various concentrations to achieve the desired [N]/[P] ratios. The formation of polyplexes, as well as the variations in their size and ζ potential, were followed by dynamic and electrophoretic light scattering. Static light scattering was utilized to assess parameters such as scattered light intensity (I_90_) and radius of gyration (R_g_). In the case of C_12_-PN-PG25, starting from [N]/[P] = 1:1, a significant reduction in both particle size and size distribution was observed as compared to the block copolymer aggregates alone (Table 1 and Table 2), implying the formation of better defined and more compact and dense particles. The further increase of [N]/[P] led to an additional reduction in particle size, reaching an average diameter of about 80 nm at the highest [N]/[P] ratio studied, [N]/[P] = 10:1 (Figure 2a, Table 2). Other indications for the formation of more compact and dense particles upon complexation are the changes in the scattered light intensity (I_90_) and the quantity R_g_/R_h_ compared to those of the initial copolymer aggregates (Figure 3a and Figure 4a). Indeed, at approximately equal total concentrations, the scattered light intensity of the polyplexes was ca. one order of magnitude higher (Figure 3a), indicating an increase in the molar mass, which was accompanied by a reduction in the particle size as noted above. The simultaneous increase in I_90_ (hence, molar mass) and size reduction indicated an increase in the density of the material within the particle. Furthermore, upon complexation, the radius of gyration, R_g_, was found to decrease more quickly and more strongly than the hydrodynamic radius, R_h_ (Table S1), which generated variations in the quantity R_g_/R_h_ (Figure 4a). The latter gives useful information on the particle density and structure [45,46]. Here, R_g_ was obtained from the partial Berry plots, whereas R_h_ was calculated from the angular dependence of the relaxation rate as described in Supplementary Materials. Representative plots are shown in Figures S1 and S2, whereas the results are collected in Table S1. As seen from Figure 4a, R_g_/R_h_ of the investigated systems dropped down from a value of about 1.2 for the initial copolymer aggregates to values in the 0.91–1.04 range for the complexes, typifying more compact structures [45,46].

The variations in the ζ potential showed an abrupt transition from highly negative to highly positive values within a very narrow [N]/[P] range with no changes upon further increase of [N]/[P] (Table 2). The strongly positive ζ potential at [N]/[P] ≥ 2.5, comparable with that of the initial copolymer aggregates (see Table 1 and Table 2), indicated the formation of polyplex particles, which apparently were not segregated into a water-insoluble core consisting of complexed oppositely charged segments of PN and DNA surrounded by PG chains building a shell. Most probably, polycation segments, not involved in the complexation with DNA, were mixed with PG chains in the outwards of the particles. Such an arrangement would satisfactorily explain the experimental results.

The behavior of the copolymer of higher PG content, C_12_-PN-PG60, in many aspects is similar to that of C_12_-PN-PG25, implying that the same events took place upon complex formation with DNA: sharp transition from strongly negative to strongly positive ζ potential values (Table 2); comparable ζ potential values of the polyplexes at higher [N]/[P] ratios with that of the initial C_12_-PN-PG60 aggregates (Table 1 and Table 2); a simultaneous increase in the scattered light intensity by order of magnitude (Figure 3b) and reduction in the size (Table 2) and quantity R_g_/R_h_ (Figure 4b) into lower values. However, some of the effects, particularly the variations in the size of polyplex particles (Figure 2b and Table 2) and, to some extent, particle size distribution (Table 2) and reduction in R_g_/R_h_ (Figure 4b), were less well pronounced. This could be attributed to the longer PG chain, which may compensate for the shrinkage of the particles upon complexation with DNA, thus making the differences less dramatic. The data obtained from dynamic, static, and electrophoretic light scattering measurements suggest that the electrostatic interactions between the oppositely charged groups of DNA and C_12_-PN-PG copolymers, leading to charge neutralization, are strong enough to convert the initial loose polymer aggregates into more hydrophobic, better defined, and more compact and dense polyplex particles.

**Figure 5:**
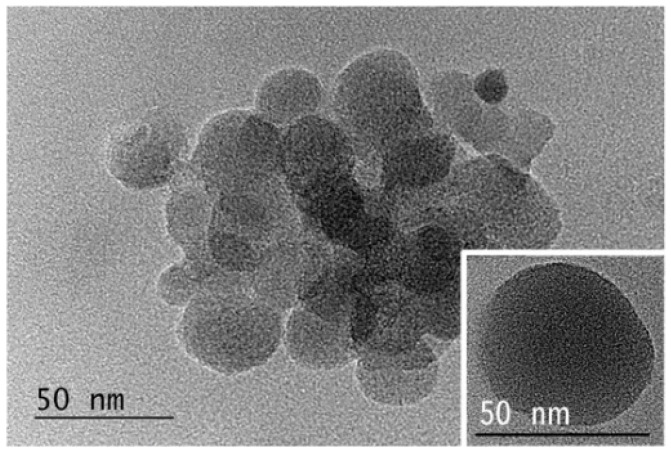
TEM images of C_12_-PN-PG25 polyplex with DNA at [N]/[P] = 10:1.

The polyplexes’ morphology was visualized by TEM. The images show clusters of aggregated spherical nanoparticles with sizes that are somewhat smaller than those obtained from DLS measurements (Figure 5). The measured smaller average sizes are most likely due to shrinkage of particles as a result of dehydration upon sample preparation. The spherical shape of the polyplexes is clearly visible from the image of an individual particle (Figure 5, inset).

### 2.3. In Vitro Cytotoxicity Assessment of Block Copolymers and Polyplexes

**Figure 6:**
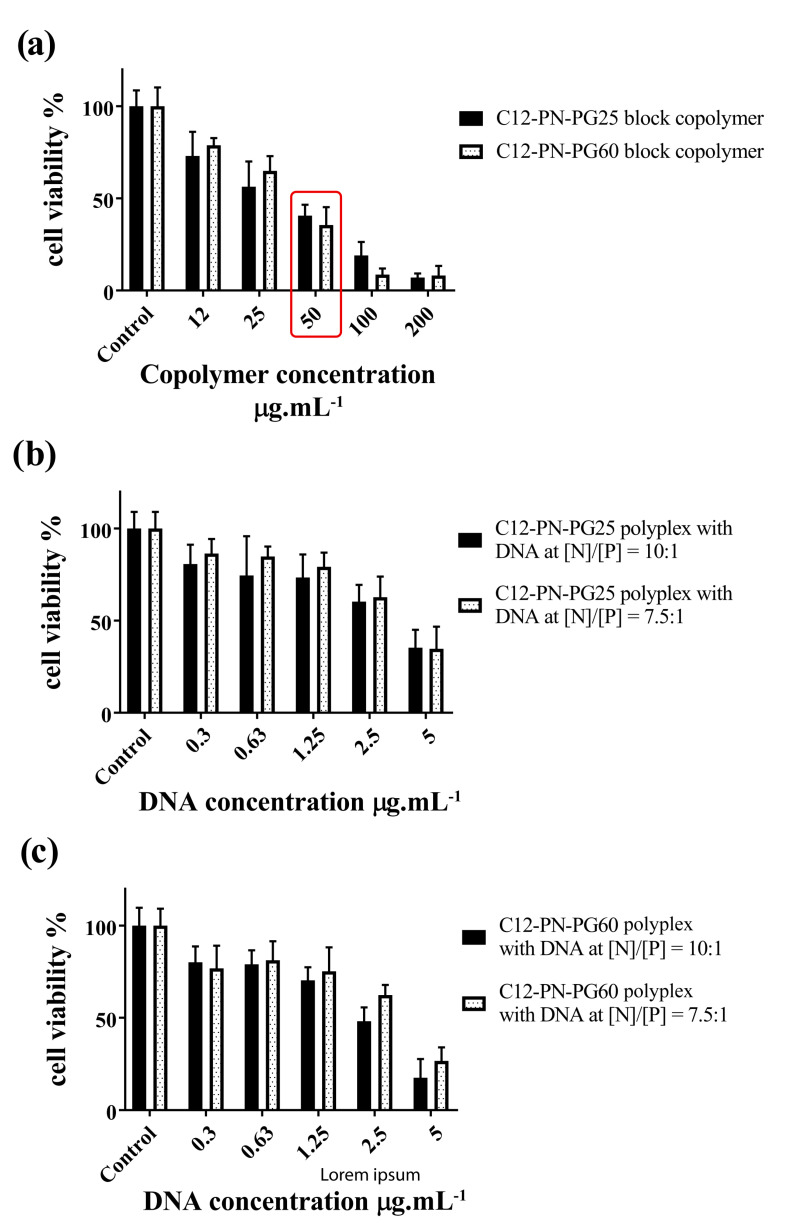
Bar graphs presenting viable cells as a percent of non-treated controls, tested by Trypan blue dye exclusion test by using automated cell counter Corning^®^ against: (**a**) concentration of block copolymers C_12_-PN-PG25 and C_12_-PN-PG60 (X axis), the red rectangle indicate the concentration of the copolymers in the highest DNA content (µg·mL^−1^) used in corresponding polyplexes; (**b**) concentration of DNA in C_12_-PN-PG25 polyplexes at [N]/[P] ratios of 10:1 and 7.5:1; (**c**) concentration of DNA in C_12_-PN-PG60 polyplexes at [N]/[P] ratios of 10:1 and 7.5:1. Each bar represents the arithmetic mean ± SD of the percent viable cells of 3 separate experiments.

The cytotoxic profile of C_12_-PN-PG25 and C_12_-PN-PG60 copolymers and the corresponding polyplexes at various [N]/[P] ratios was evaluated in order to estimate the potential harmful effects. This test is very important as the polymers utilized so far as non-viral vectors for gene therapy demonstrate noticeable cytotoxicity, which is one of the main drawbacks. The cytotoxicity of non-viral vectors depends on a number of physicochemical parameters such as particle size, morphology, and ζ potential that can also affect the gene delivery process [47]. The experiments were performed with three human cell lines, *A549* (lung adenocarcinoma), *MCF-7* (breast cancer), and *HeLa* (cervical cancer), that were exposed to different concentrations of pure copolymers and their corresponding polyplexes for 72 h. Cell viability was assessed by 3-(4,5-dimethylthiazol-2-yl)-2,5-diphenyltetrazolium salt (MTT). The calculated half-maximal inhibitory concentration (IC_50_) of C_12_-PN-PG25 and C_12_-PN-PG60 copolymers and corresponding polyplexes at various [N]/[P] is presented in Table 3. The *HeLa* cell line was further analyzed for more precise determination of cytotoxicity by another approach—a Trypan blue dye exclusion test. The pure copolymers were tested in a wider concentration range—from 12.5 to 200 µg·mL^−1^. The results are presented in Figure 6a. As seen, they are similar to those obtained by the MTT analysis.

The direct comparison of the IC_50_ values of the two copolymers showed slightly higher cytotoxicity for C_12_-PN-PG60 for two of the cell lines (Table 3). This was a somewhat surprising finding since the lower toxicity and higher biocompatibility of C_12_-PN-PG60 were anticipated due to its longer polyglycidol chains and higher polyglycidol content. The results can be rationalized in terms of similarities in the physicochemical parameters and derived structure of the aggregates of the two copolymers (Table 1): relatively large in size, loose particles, exhibiting strongly positive ζ potential and dimensions differing by less than 6%.

The IC_50_ values of the polyplexes of the two copolymers were found to generally increase with increasing [N]/[P]. This trend could also be correlated with the way polyplex particles change their dimensions, structure, and physicochemical parameters. As shown in the previous section, more compact and smaller in size structures are formed at higher [N]/[P] ratios. Apparently, the compact structure and smaller dimensions of the polyplex particles are beneficial as far as cytotoxicity and biocompatibility are concerned.

Considering the favorable characteristics of the polyplex particles at elevated [N]/[P] ratios, the polyplexes prepared at [N]/[P] ratios of 7.5:1 and 10:1 were further evaluated by a Trypan blue dye exclusion test on the *HeLa* human cell line. For these experiments, we chose a concentration range of DNA comparable with those regularly used in transfection experiments, namely, from 0.5 to 2.5 µg·mL^−1^ [48,49]. The results presented in Figure 6b,c showed low to moderate toxicity of the polyplexes. Only at a concentration of DNA of 5 µg·mL^−1^, which is outside the concentration range, in which DNA is typically used in transfection experiments [48,49], the cell viability was below IC_50_ (Figure 6b,c).

### 2.4. Cell Internalization of Polyplexes

**Figure 7:**
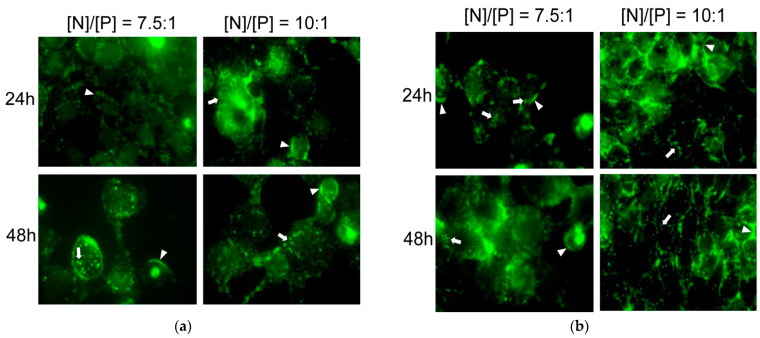
Fluorescent microscopy of *HeLa* cells, incubated for 24 or 48 h with C_12_-PN-PG25 and DNA (**a**) and C_12_-PN-PG60 and DNA (**b**) polyplexes at [N]/[P] ratios of 10:1 and 7.5:1. All images within an experiment were taken under identical settings. The thick arrows show clustered internalized polyplex nanoparticles. The arrowheads show structures most probably located around the cells stacked to the cellular membrane. The thin arrows show secondary staining of the cellular DNA due to some release of the dye.

To investigate the cellular uptake of the resulting polyplexes into the cells, SYBR Green-stained DNA (~2000 bp salmon sperm DNA) was used for complex formation. SYBR green is a commonly used cell-permeable fluorescent dye that intercalates non-specifically into double-stranded DNA. Upon interaction with double-stranded DNA molecules, the brightness of this fluorescent dye strongly (>1000-fold) increases. Prior to incubation, the salmon DNA was ethanol precipitated and re-dissolved in order to avoid SYBR Green staining of the cellular DNA. The results of microscopic observations are presented in Figure 7. It is evident that both copolymers were able to introduce DNA into cells. The effect was anticipated and attributed to the strong positive surface charge of the polyplex particles and their relatively small size and compact structure (Table 2, Figure 4, Section 2.2).

An increased cell penetration was observed 48 h after inoculation with complexes of both copolymers, indicating that (i) they were stable for at least 48 h in physiological conditions and (ii) longer time for internalization was needed. In the case of C_12_-PN-PG25 polyplexes, there was no visible difference in the level of penetration for both [N]/[P] ratios (Figure 7a, bottom line). In contrast, the level of penetration of the C_12_-PN-PG60 polyplexes at the [N]/[P] ratio of 7.5:1 was much more prominent (Figure 7b, bottom left image) than that at 10:1, implying that polyglycidol above certain critical length and/or content may have an effect of hindering of cellular uptake.

## 3. Materials and Methods

### 3.1. Materials and Reagents

All chemicals were purchased from Sigma-Aldrich. N,N-Dimehylformamide (DMF, ≥99.5%) was distilled from calcium hydride prior to use. α,α′-Azoisobutyronitrile (AIBN, 98%) was recrystallized from methanol. The 2-Aminoethanethiol hydrochloride (AET·HCl, ≥98%) was dried in a vacuum prior to use. A stock solution of 100 µg·mL^−1^ salmon sperm DNA (2000 bp, M_w_ ≈ 1.3 × 10^6^ Da) was prepared in ultrapure water (>18 MΩ·cm) and used for complex formation. Well-defined amphiphilic block copolymers with pendant alkene and hydroxyl functional groups, as well as a dodecyl residue at the end of the chain (C_12_-PAGE-PG25 and C_12_-PAGE-PG60), were prepared by sequential anionic ring-opening polymerization of allylglycidyl ether (AGE) and ethoxyethyl glycidyl ether (protected glycidol) and subsequent cleavage of the protective groups as previously described [42]. The composition, molecular weights, and codes of the copolymer precursors are presented in Table 1.

### 3.2. Synthesis of Amine and Hydroxyl-Functional Copolyethers (C_12_-PN-PG)

Typically, the amphiphilic block copolymer C_12_-PAGE-PG25 with composition C_12_H_25_-(AGE)_44_-(G)_16_ (0.3 g, 2 mmol alkene groups), AIBN (0.125 g, 0.76 mmol), and AET·HCl (1.14 g, 10 mmol) were dissolved in 0.5 mL of freshly distilled DMF. The reaction mixture was degassed by bubbling argon for 30 min, and the reaction vessel was immersed into a preheated to 85 °C oil bath. The reaction proceeded at that temperature for 96 h. The mixture was diluted with distilled water, and the product was purified through ultrafiltration (membrane molecular weight cut-off: 1000 Da). The modified copolymer was recovered through lyophilization. Yield: 0.42 g, (79%). ^1^H NMR (600 MHz, DMSO-d_6_, δ, ppm): 0.86 (C***H*_3_**), 1.24 (CH_3_-(C***H*_2_**)**_10_**-), 1.76 (-O-CH_2_-C***H*_2_**-CH_2_-), 2.58 (-C***H*_2_**-S-CH_2_-), 2.76 (-CH_2_-S-C***H*_2_**-CH_2_-), 2.95 (-S-CH_2_-C***H*_2_**-NH_3_^+^), 3.25–3.75 (CH_3_-(CH_2_)_10_-C***H*_2_**-O) + -O-C***H*_2_**-CH-O- + -O-CH_2_-C***H***-O- + -CH(C***H*_2_**-O-CH_2_-) + -CH(CH_2_-O-C***H*_2_**-) + -O-C***H*_2_**-CH(-CH_2_-OH)-O- + CH_2_-C***H***-(CH_2_-OH)-O- + CH_2_-CH(-C***H*_2_**-OH)-O-).

### 3.3. Characterization

The ^1^H NMR spectra were recorded in DMSO-d_6_ on a Bruker Avance II+ 600 MHz instrument. The size distribution of the block copolymer aggregates was determined by dynamic light scattering (DLS) using a NanoBrook Plus PALS instrument (Brookhaven Instruments), equipped with a 35 mW solid-state laser operating at λ = 660 nm at a scattering angle of 90°. The particles’ hydrodynamic diameters (d_h_) were determined according to the Stokes–Einstein equation:

*d_h_ = kT*/(3*πηD*) (1)

where *k* is the Boltzmann’s constant, *T* is the absolute temperature, *η* is the solvent viscosity, *D* is the diffusion coefficient.

The *ζ* potentials were calculated from the obtained electrophoretic mobility by the Smoluchowski equation:

*ζ* = 4*πηµ/ε* (2)

where *η* is the solvent viscosity, *µ* is the electrophoretic mobility, and *ε* is the dielectric constant of the solvent. The size and *ζ* potential measurements were carried out in an automated mode in triplicate and recorded as averages of 3 and 20 runs, respectively. The copolymers’ and polyplexes’ dispersions were passed through Millipore^®^ 0.45 μm pore-sized Nylon syringe filters prior to measurements. Transmission electron microscopy (TEM) images were obtained using HRTEM JEOL JEM-4-2100 (200 kV) instrument equipped with CCD camera GATAN Orius 832 SC1000 and GATAN Microscopy Suite Software. The samples were prepared by depositing a drop of the polyplex solution onto a carbon grid and subsequent evaporation of the solvent. The images analysis was performed with ImageJ software.

### 3.4. DNA Condensation

Block copolymer aggregates were prepared by applying the direct dissolution method. A predetermined copolymer amount was dispersed in 1 mL of ultrapure water (18.2 MΩ·cm). An equal volume of DNA solution (100 µg·mL^−1^) was added dropwise under vigorous stirring (900 rpm, 2 min) at room temperature. The molar ratio between the positively charged groups of the block copolymers and the phosphate groups of the DNA ([N]/[P]) was varied from 1:1 to 10:1. The electrostatically formed complexes were gently stirred (150 rpm) at room temperature for 30 min and subjected to characterization.

### 3.5. Cell Lines and Culture Conditions

The cytotoxicity of amine-functionalized copolymers (C_12_-PN-PG25 and C_12_-PN-PG60) and the corresponding polyplexes at various [N]/[P] ratios were tested against three human cell lines. *MCF-7* (breast cancer cell line) cells were grown in Eagle’s Minimum Essential Medium (Thermo Fisher Scientific, Waltham, MA, USA) with 0.01 mg·mL^−1^ human recombinant insulin (Sigma-Aldrich, Milwaukee, WI, USA), 10% fetal bovine serum (Thermo Fisher Scientific, Waltham, MA, USA), and penicillin-streptomycin (Thermo Fisher Scientific, Waltham, MA, USA). *A549* (lung adenocarcinoma cell line) cells were plated in F-12K Medium (Thermo Fisher Scientific, Waltham, MA, USA) supplemented with 10% (*v*/*v*) fetal calf serum (FCS, Thermo Fisher Scientific, Waltham, MA, USA) and 1% penicillin/streptomycin solution (Thermo Fisher Scientific, Waltham, MA, USA). *HeLa* (cervical cancer) cells were plated in Dulbecco’s modified Eagle’s medium (DMEM, Gibco BRL, Waltham, MA, USA) supplemented with 10% (*v*/*v*) fetal calf serum (FCS, Life Technologies, Inc.) and 1% penicillin/streptomycin solution (Life Technologies, Inc., Waltham, MA, USA). All cell lines used in this study were purchased from ATCC (LGC STANDARDS, Teddington, UK). The cells were maintained in a 5% CO_2_ incubator at 37 °C. Only cells growing in the exponential phase were used for all experiments.

### 3.6. Cytotoxicity Assessment (Trypan Blue Exclusion Test)

The dye exclusion test to determine the percentage of viable cells was performed as described elsewhere [50]. In this assay, a cell suspension was mixed with Trypan blue dye and then examined to determine whether cells take up (death cells) or exclude the dye (live cells) by using automated cell counter Corning^®^. The percentage of viable cells was calculated according to the formula:

viable cells (%) = (total number of viable cells per mL of aliquot)/(total number of cells per mL of aliquot) × 100(3)

### 3.7. Cytotoxicity Assessment (MTT-Dye Reduction Assay)

The cell viability was assessed using the standard MTT-dye reduction assay, as described previously [51], with some modifications [23]. The method is based on the biotransformation of the yellow tetrazolium salt MTT (3-(4,5-dimethylthiazol-2-yl)-2,5-diphenyltetrazolium bromide) to a violet formazan via the mitochondrial succinate dehydrogenase in the viable cells. Briefly, the exponentially growing cells were seeded in 96-well flat-bottomed micro-plates (Corning Costar Flat Bottom Cell Culture Plate) at a density of 1 × 10^4^ cells per mL, 100 µL per well. After 24 h of incubation at 37 °C, the cells from each cell line were treated with the C_12_-PN-PG25 and C_12_-PN-PG60 copolymers and the corresponding polyplexes at various [N]/[P] ratios. After a 72 h incubation at conditions of 5% CO_2_ at 37 °C, the medium was changed to a phenol red-free medium, and MTT (Invitrogen) was added in a final concentration of 0.5 mg·mL^−1^. The cells were incubated for 2 h at conditions of 5% CO_2_ and 37 °C. Finally, 100 µL of DMSO per well were added to dissolve the formed formazan crystals. The measurement of the absorbance of the samples was performed on a Varioskan LUX Multimode Microplate Reader (Thermo Fisher Scientific) at 580 nm. GraphPad Prism software v.8 was used for data analysis.

### 3.8. Intracellular Localization

Salmon sperm DNA fragments (2000 bp) were pre-stained with SYBR green dye (Thermo Fisher Scientific) and then purified from the free dye by column filtration prior to use. Polyplexes were prepared at different [N]/[P] ratios varying from 1:1 to 10:1. The cells used in these experiments were plated at a density of 1 × 10^5^ in 24-well dishes and cultivated for 24 h prior to the incubation with corresponding pre-stained polyplexes for 2 or 24 h. Images were obtained with a Zeiss Axiovert 200 M microscope using an Objective LD Plan-Neofluar 63x/0.75 Corr (D = 0–1.5 mm), equipped with a CCD camera AxioCam MRm driven by Axiovision v4.9 software (Carl Zeiss Microscopy LLC, White Plains, NY, USA), as described previously [23]. Three independent experiments were performed.

## 4. Conclusions

Radical addition of 2-aminoethanethiol hydrochloride to the allylic double bonds of two related in composition block copolymers of poly(allyl glycidyl ether) and polyglycidol was employed to orthogonally introduce pendant primary amine groups. The modification reaction proceeded with high efficiency to yield 100% functionalized copolymers. The introduction of the amine groups, turning the moderately hydrophobic PAGE blocks into strongly hydrophilic ones, did not prevent the aggregation of the copolymers: in aqueous solution, they formed relatively large, multichain, strongly positively charged and loose aggregates, held together by hydrophobic interactions between the dodecyl residues and hydrogen bonding from the polyglycidol moieties. These aggregates had the capacity to efficiently condense DNA via electrostatic interactions, thereby forming compact and dense polyplex particles. Although strongly positively charged at [N]/[P] ratios of above 2, the latter exhibited low to moderate toxicity, which was attributed to the biocompatibility of polyglycidol. The strongly positive ζ potential of the polyplex particles favored the interactions with the negatively charged cell membranes, which resulted in enhanced cell penetration. The polyplexes remained stable at physiological conditions for an extended period of 48 h, which can be related to additional stabilization of the polyplex structures via hydrogen bonding between the hydroxyl groups of the polyglycidol moieties. The polyglycidol content had no or only a little effect on the physicochemical characteristics of the initial aggregates, polyplex particles, and their biological performance, implying that polyglycidol content in the 25–60 mol % range is optimal. Overall, the results demonstrated the potential of appropriately modified polyglycidol-based copolymers as non-viral vectors for gene delivery.

## Figures and Tables

**Table 1:** Characteristics of block copolymer precursors, amine-functional copolymers, and the corresponding aggregates in aqueous media.

Precursors					Aggregates of Modified Block Copolymers			
*Code*	*DP* ^a^ *_PAGE_*	*DP* ^a^ *_PG_*	*M_n_*^a^ (g·mol^−1^)	*Ð* *_M_* ^b^	*Code*	*d_h_*^c^ (nm)	*PdI* ^c^	*ζ* ^c^ (mV)
C_12_-PAGE-PG25	44	16	6400	1.08	C_12_-PN-PG25	184.2	0.293	41.1
C_12_-PAGE-PG60	44	66	10,100	1.06	C_12_-PN-PG60	173.9	0.232	40.7

^a^ Number-average degrees of polymerization (*DP*) and molar masses (*M_n_*), as determined by ^1^H NMR analyses. ^b^ Molar-mass dispersity (*Ð_M_*), as determined before the deprotection of the PG-hydroxyl groups by GPC in THF vs. polystyrene standards. ^c^ Average diameters (*d_h_*), polydispersity indexes (*PdI*, *distribution of particle size*), and zeta potentials (*ζ*) of the modified block copolymer aggregates in aqueous media, as determined by DLS and electrophoretic light scattering. ^c^ Standard deviations for d_h_ and ζ are ±2% and ±3%, respectively.

**Table 2:** Characteristics from DLS and electrophoretic LS measurements of polyplexes from C_12_-PN-PG25 and C_12_-PN-PG60 at various [N]/[P] ratios in aqueous media.

[N]/[P]	*d_h_*^a^ (nm)	*PdI*	*ζ*^a^ (mV)
***Polyplexes from C*_12_*-PN-PG25 and DNA***			
1:1 2.5:1 5:1 7.5:1 10:1	127.6	0.108	−25.7
	112.8	0.153	37.4
	95.4	0.073	35.3
	92.0	0.113	34.2
	78.7	0.135	35.7
***Polyplexes from C*_12_*-PN-PG60 and DNA***			
1:1 2.5:1 5:1 7.5:1 10:1	181.1	0.090	−27.9
	131.2	0.090	36.8
	119.5	0.187	40.7
	100.8	0.148	38.9
	126.6	0.191	41.8

^a^ Standard deviations for *d_h_* and *ζ* are ±2% and ±3%, respectively.

**Table 3:** Calculated half-maximal inhibitory concentration (IC_50_) in µg·mL^−1^ of C_12_-PN-PG25 and C_12_-PN-PG60 copolymers and corresponding polyplexes at various [N]/[P] ratios.

[N]/[P]	*A549*	*MCF-7*	*HeLa*
***Copolymer aggregates and polyplexes from C*_12_*-PN-PG25 and DNA***			
Initial C_12_-PN-PG25 aggregates 1:1 2.5:1 5:1 7.5:1 10:1	32.2 ± 5.1	29.8 ± 3.5	27.7 ± 2.8
	20.7 ± 1.8	26.2 ± 3.1	31.5 ± 3.6
	24.7 ± 2.2	28.8 ± 2.6	24.6 ± 5.1
	37.2 ± 3.4	47.2 ± 3.3	53.7 ± 4.7
	46.5 ± 2.8	39.8 ± 4.1	52.8 ± 3.4
	49.8 ± 3.1	48.2 ± 2.5	43.7 ± 2.9
***Copolymer aggregates and polyplexes from C*_12_*-PN-PG60 and DNA***			
Initial C_12_-PN-PG60 aggregates 1:1 2.5:1 5:1 7.5:1 10:1	22.0 ± 4.4 *	24.6 ± 4.2 *	28.7 ± 3.2
	35.5 ± 2.7	19.8 ± 4.6 *	32.6 ± 3.5
	40.3 ± 1.9	25.6 ± 2.8	39.5 ± 4.8
	44.2 ± 2.6	49.5 ± 3.5	56.6 ± 3.7
	50.1 ± 3.3	46.7 ± 3.7	48.7 ± 4.2
	48.8 ± 4.1	42.2 ± 3.9	43.8 ± 2.9

Each data point represents the arithmetic mean ± SD of 3 independent experiments performed in octuplicates. Statistical significance was calculated by ordinary one-way ANOVA, followed by Tukey’s multiple comparisons test, and * *p*-values ≤ 0.05 were considered to indicate statistical significance as these analyses of variances did not find significant differences between the groups.

## Supplementary Materials

The following are available online at https://www.mdpi.com/article/10.3390/ijms22179606/s1, Figure S1: Relaxation rate (Γ) as a function of sin^2^(θ/2) for aqueous dispersions of polyplex particles prepared from (a) C_12_-PN-PG25 and DNA at [N]/[P] = 5.0:1 and (b) C_12_-PN-PG60 and DNA at [N]/[P] = 7.5:1. Figure S2: Partial Berry plots for determination of R_g_ of polyplex particles prepared from (a) C_12_-PN-PG25 and DNA at [N]/[P] = 10.0:1 and (b) C_12_-PN-PG60 and DNA at [N]/[P] = 5.0:1. Table S1: Static and dynamic light scattering parameters of the initial block copolymer aggregates and polyplexes with DNA at various [N]/[P] ratios in aqueous media.
