# Supplementary material for: Functional Polyglycidol-Based Block Copolymers for DNA Complexation
Source: Int J Mol Sci. 2021 Sep 4;22(17):9606. doi: 10.3390/ijms22179606 (PMC8431755; doi:10.3390/ijms22179606)
Supplement: Supplementary file 1 [file ijms-22-09606-s001.zip › ijms-1334851-supplementary.pdf]

Supplementary Materials

Functional Polyglycidol-Based Block Copolymers for DNA Complexation

Radostina Kalinova [1], Miroslava Valchanova [2], Ivaylo Dimitrov [1,*], Sevdalina Turmanova [2], Iva Ugrinova [3,*], Maria Petrova [3], Zlatina Vlahova [3] and Stanislav Rangelov [1,*]

[1] Institute of Polymers, Bulgarian Academy of Sciences, Sofia, Bulgaria
[2] Department of Material Science and Technology, University “Prof. Assen Zlatarov”, Burgas, Bulgaria
[3] Institute of Molecular Biology, Bulgarian Academy of Sciences, Sofia, Bulgaria

* Correspondence: rangelov@polymer.bas.bg (S.R.); dimitrov@polymer.bas.bg (I.D.); ugryiva@gmail.com (I.U.)

Dynamic light scattering (DLS). DLS measurements were performed on a Brookhaven BI-200 goniometer with vertically polarized incident light at a wavelength λ = 633 nm supplied by a He–Ne laser operating at 35 mW and equipped with a Brookhaven BI-9000 AT digital autocorrelator. Measurements were made at angles θ in the 50 – 130° range. The autocorrelation functions were analyzed using the constrained regularized algorithm CONTIN [1] to obtain the distributions of the relaxation rates (Γ). The latter provided distributions of the apparent diffusion coefficient ($D = \Gamma/q^2$) where q is the magnitude of the scattering vector given by $q=(4\pi n/\lambda)\sin(\theta/2)$, n is the refractive index of the medium. The mean hydrodynamic radius was obtained by the Stokes–Einstein equation (1):

$$R_h = kT/(6\pi\eta D) \qquad (1)$$

where k is the Boltzmann constant, η is the solvent viscosity at temperature T in Kelvin and D is the diffusion coefficient. The diffusion coefficients were determined from the slopes of the linear fit of the data plotted as relaxation rate versus $\sin^2(\theta/2)$. Representative Γ versus $\sin^2(\theta/2)$ plots are shown in Figure S1. The results for D and R_h are collected in Table S1. All measurements were performed at 25 °C at a single solute concentration. The initial dispersions were

additionally diluted by adding varying quantities of water to get equal total solute (copolymer and DNA) concentrations. The standard deviation of the method is ±2%.

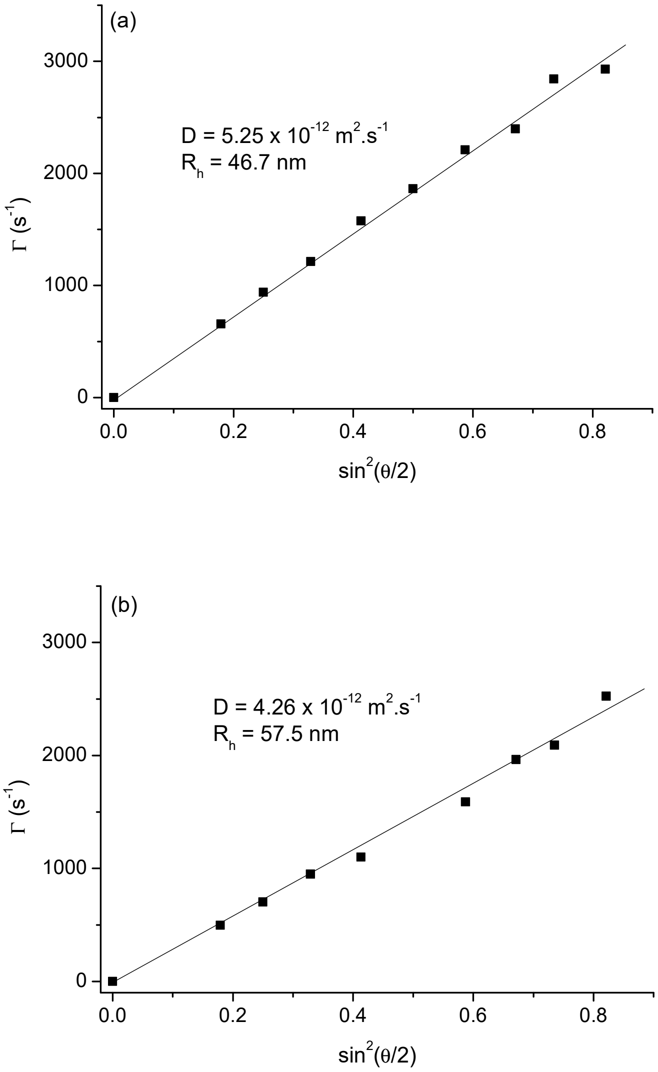

Figure S1. Relaxation rate (Γ) as a function of $\sin^2(\theta/2)$ for aqueous dispersions of polyplex particles prepared from (a) C_{12}-PN-PG25 and DNA at [N]/[P] = 5.0:1 and (b) C_{12}-PN-PG60 and DNA at [N]/[P] = 7.5:1.

Static light scattering (SLS). The SLS measurements were carried out in the interval of angles from 40 to 140° using a Brookhaven BI-200 goniometer with vertically polarized incident light at a wavelength λ = 633 nm supplied by a He–Ne laser operating at 35 mW. The radii of

gyration, R_g, were obtained by partial Berry plots from the dependences of $I'^{-1/2}$ on q^2, where I' is the quantity $I_{ex}\sin\theta$, with I_{ex} being the excess of scattered light intensity, and q^2 is the scattering vector defined above. Representative partial Berry plots are shown in Figure S2. The results for R_g are collected in Table S1. All measurements were performed at 25 °C at a single solute concentration. The initial dispersions were additionally diluted by adding varying quantities of water to get equal total solute (copolymer and DNA) concentrations. The standard deviation of the method is ±2%.

I_{90} was assessed from the static light scattering data from the excess of the scattered light intensity at an angle of 90°.

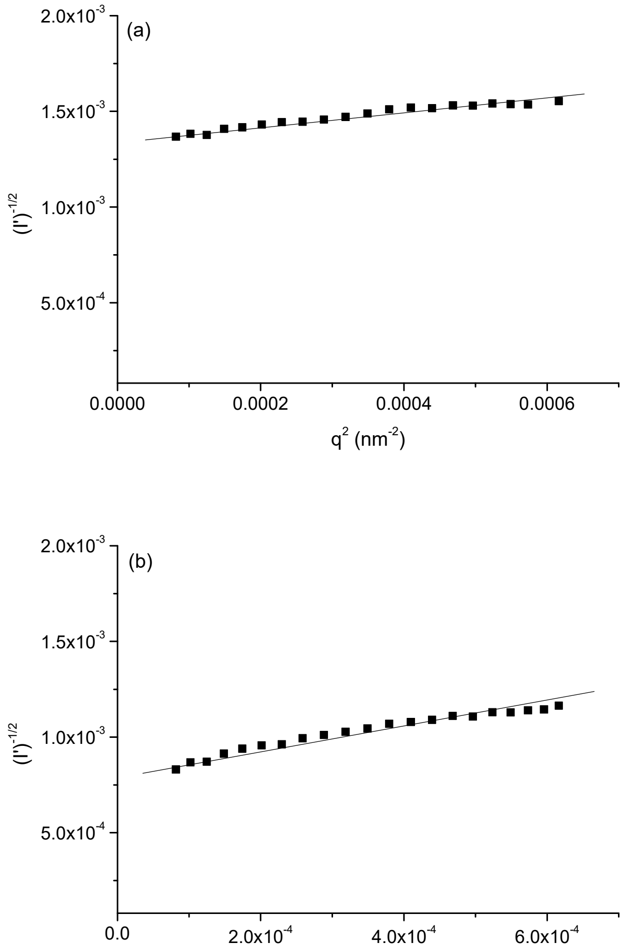

Figure S2. Partial Berry plots for determination of R_g of polyplex particles prepared from (a) C_{12}-PN-PG25 and DNA at [N]/[P] = 10.0:1 and (b) C_{12}-PN-PG60 and DNA at [N]/[P] = 5.0:1.

Table S1. Static and dynamic light scattering parameters of the initial block copolymer aggregates and polyplexes with DNA at various [N]/[P] ratios in aqueous media. The standard deviations are ±2%.

[N]/[P]	10^{12} x D (m^2 s^{-1})	R_h (nm)	R_g (nm)
Polyplexes from C_{12}-PN-PG25 and DNA			
Initial ***C_{12}-PN-PG25*** aggregates	3.38	72.5	85.4
1.0:1	-	-	-
2.5:1	3.82	64.2	58.7
5.0:1	5.25	46.7	48.8
7.5:1	5.04	48.6	46.0
10:1	5.76	42.6	39.7
Polyplexes from C_{12}-PN-PG60 and DNA			
Initial ***C_{12}-PN-PG60*** aggregates	3.28	74.7	108.0
1.0:1	3.04	80.6	107.5
2.5:1	4.38	56.0	69.2
5.0:1	4.44	55.2	64.6
7.5:1	4.26	57.5	55.4
10:1	-	-	-

Reference

1. Provencher, S.W. Inverse problems in polymer characterization: Direct analysis of polydispersity with photon correlation spectroscopy. *Macromol. Chem.* **1979**, *180*, 201–209. DOI: 10.1002/macp.1979.021800119
